# The Prevalence and Impact of Urinary Incontinence on Multiple Sclerosis Patients in Taif City, Saudi Arabia

**DOI:** 10.7759/cureus.57010

**Published:** 2024-03-26

**Authors:** Adnan A Mubaraki, Matooqa A Alnemari, Sarah O Aljuaid, Fai M Altalhi, Yazan M Alamri, Shahad O Altowairqi

**Affiliations:** 1 Medicine, Taif University, Taif, SAU; 2 Medicine and Surgery, Taif University, Taif, SAU

**Keywords:** multiple sclerosis, lower urinary tract symptoms, impact, prevalence, urine incontinence

## Abstract

Introduction

Multiple sclerosis (MS) is a chronic inflammatory disease that results in demyelination and progressive loss of nerve cells within the central nervous system.

Multiple sclerosis, as well as other neurological diseases that impact brain structures and spinal pathways involved in sphincter control, may cause lower urinary tract symptoms (LUTS)

Our aims are to determine the prevalence, severity, and impact on the quality of life of urinary incontinence among MS patients in Taif, Saudi Arabia, as well as its potential association with demographics and clinical features.

Method

A cross-sectional study included 150 of MS patients aged 18 years and older who completed the validated Arabic versions of both the International Consultation on Incontinence Questionnaire Overactive Bladder (ICIQ-OAB) and the International Consultation on Incontinence Questionnaire-Urinary Incontinence Short Form (ICIQ-UI SF). In addition, other clinical parameters were collected from the medical records of patients.

The data were analyzed using Statistical Package for the Social Sciences (SPSS) version 26 (IBM Inc., Armonk, New York). Qualitative data were expressed as numbers and percentages, and the Chi-squared test (χ2) was employed. Quantitative data were expressed as means with standard deviation (mean ± SD).

Result

67.3% of the participants were female; the mean ICIQ UI and ICIQ OAB scores in these MS patients were found to be 6.30 ± 6.26 and 5.32 ± 3.76, respectively. They were significantly higher in progressive MS patients compared to relapsing-remitting MS patients (p<0.05). There was a high positive correlation obtained between ICIQ UI and ICIQ OAB scores (rho=0.801, p<0.001).

Conclusion

The findings of this study showed that urinary incontinence was a common and distressing symptom experienced by individuals with MS. The severity of UI symptoms was significantly more in progressive multiple sclerosis compared to relapsing-remitting multiple sclerosis.

## Introduction

Multiple sclerosis (MS) is a chronic inflammatory disease resulting in demyelination and progressive loss of nerve cells within the central nervous system.

In MS impact will be on myelin sheath of the brain and spinal cord that involved in sphincter control that may cause lower urinary tract symptoms (LUTS) [1].

MS is the most prevalent neurological disease in 20 to 45-year-olds, and it is three times less prevalent in men than in women. It usually presents complications resulting from partial remission, with one of the possible complications being urinary disorders, affecting 50-80% of patients. In 70% of these patients, the urinary symptoms prognosis is associated with functional and neurological deterioration. The most frequent symptoms are urinary retention, urgency, nocturia, polyuria, and incontinence. The presence of urinary tract infections (UTIs) in MS patients is associated with difficulty emptying the bladder [2].

A total of 65% of MS patients have moderate to severe urinary symptoms, and up to 14% initially present with urinary symptoms. Urinary retention, neurogenic detrusor overactivity, and detrusor sphincter dyssynergia increase the chance of urinary tract infections in patients with MS, and these infections may exacerbate the immune response, leading to advanced symptoms [3].

Because MS patients have urinary problems, they are at risk of urinary tract colonization, leading to urinary tract infections. Risk factors, such as high bladder pressure, increased urinary stasis, bladder stones, and catheters, are associated with neurogenic bladder UTIs.

According to the 2005 North American Research Committee on Multiple Sclerosis (NARCOMS), 16,858 surveys were distributed, and only 9702 (58%) were completed.

Sixty-five percent of participants with MS reported at least one moderate to severe urinary symptom, which was correlated with longer disease duration [4]. These data have not been confirmed in a more recent cohort, and there remains a lack of data on the nature of stress urinary incontinence (SUI) in patients with MS. Despite the high prevalence of urinary symptoms in the population, women with MS are most likely to seek treatment for overactive bladder, complaining of urinary urgency, frequency, and urge urinary incontinence (UUI) [5].

In this study, we aimed to determine the prevalence and severity of UI and its impact on the quality of life (QOL) of MS patients in Taif, Saudi Arabia, as well as its potential association with demographics and clinical features.

## Materials and methods

A cross-sectional study was conducted from September 2022 to June 2023 at Alhada Armed Forces Hospital, King Abdulaziz Specialist Hospital, and King Faisal Medical Complex in Taif, Saudi Arabia. This study was approved by the Armed Forces Hospital Institutional Review Board (H-02-T-078) and the Health Affairs Directorate (HAP-02-T-067).

The inclusion criteria for this study were patients aged 18 years and older with a confirmed MS diagnosis. Patients younger than 18 years old, those unable to provide informed consent, or those taking medications that could potentially affect urinary system function were excluded. 

During routine visits to a neurology clinic, the patients were asked to complete the validated Arabic versions of the International Consultation on Incontinence Questionnaire Overactive Bladder (ICIQ-OAB) [6] questionnaire and the International Consultation on Incontinence Questionnaire-Urinary Incontinence Short Form (ICIQ-UI SF) [7]. These questionnaires include questions regarding UI, leakage amount, and impact on the QOL (scoring scale 0-21), where the highest number indicates the greatest impact. The ICIQ-OAB questionnaire addresses the impact of urinary frequency, urgency, urge incontinence, and nocturia on the QOL (scoring scale 0-16), with higher scores indicating more severe symptoms. Furthermore, the patients who agreed to participate in the study had their medical records reviewed for clinical parameters, including age, gender, smoking status, comorbidities, type and duration of MS, and use of disease-modifying therapies.

Statistical analysis

The data were analyzed using Statistical Package for the Social Sciences (SPSS) version 26 (IBM Inc., Armonk, New York). Qualitative data were expressed as numbers and percentages to test the relationship between variables, and the Chi-squared test (χ2) was employed. Quantitative data were expressed as means with standard deviation (mean ± SD). A univariate analysis of the covariance (ANCOVA) model was used to assess the influence of patient variables on ICIQ UI and ICIQ OAB. A p-value of less than 0.05 was considered statistically significant.

## Results

The analysis included data regarding UI from 150 MS patients. Sociodemographic details showed that 101 participants (67.3%) were females, 71 (47.3%) had a bachelor's degree, 102 (68%) were residents of the Taif region, and only 27 (18%) were smokers (Table 1). The mean ICIQ UI and ICIQ OAB scores for these MS patients were 6.30 ± 6.26 and 5.32 ± 3.76, respectively.

**Table 1:** Sociodemographic characteristics of the MS patients in this study MS - multiple sclerosis

Variables		Number	Percentage
Gender	Female	101	67.3
	Male	49	32.7
Education level	No primary education	3	2
	Primary	8	5.3
	Middle	13	8.7
	Secondary	42	28
	Graduate	71	47.3
	Masters or higher	13	8.7
Smoker	No	123	82
	Yes	27	18

**Figure 1:**
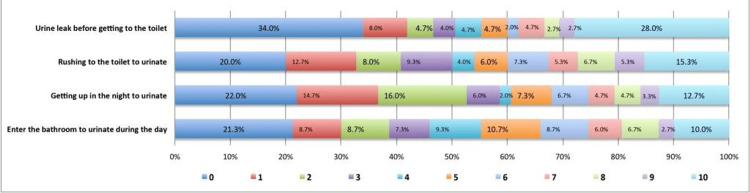
Urinary incontinence symptoms in MS patients MS - multiple sclerosis

The distribution of various UI symptoms is shown in Figure 1. The severity of the symptoms was measured on a 10-point scale. The most commonly reported UI symptom was "urine leak before getting to the toilet", followed by "rushing to the toilet to urinate", "getting up at night to urinate", and "going to the bathroom to urinate during the day".

The mean ICIQ UI score was significantly higher for patients older than 46-55 years (10.52±6.20) and ≥56 years (10.0±6.72) than for the other age groups (p<0.001). The mean ICIQ UI values were significantly higher in widowed patients (p<0.001) and those who had education at the middle school level or lower (p=0.005). The gender, educational level, residence, and smoking status of the patients did not show any statistical differences in the mean ICIQ UI scores (Table 2).

**Table 2:** Comparison of ICIQ UI scores based on different sociodemographic characteristics * A p-value <0.05 is considered statistically significant ICIQ UI - International Consultation on Incontinence Questionnaire-Urinary Incontinence

Variables		Number of patients	Mean	Standard deviation	p-value*
Age	≤25 years	18	2.67	4.653	<0.001
	26–35 years	58	4.78	5.532	
	36–45 years	41	6.73	6.253	
	46–55 years	27	10.52	6.204	
	≥56 years	6	10.00	6.723	
Gender	Female	101	6.55	6.120	0.477
	Male	49	5.78	6.584	
Education level	No primary education	3	11.67	10.214	0.005
	Primary	8	9.50	7.051	
	Middle	13	10.69	4.733	
	Secondary	42	6.98	6.653	
	Graduate	71	4.85	5.781	
	Masters or higher	13	4.46	4.409	
Smoker	No	123	6.40	6.307	0.683
	Yes	27	5.85	6.156	

The mean ICIQ OAB scores were also found to be significantly higher for patients older than 45 years (p<0.001), widowed patients (p=0.001), and those who had education at the middle school level or lower (p<0.001). However, the gender, residence, and smoking status of the patients did not show any statistical differences in the ICIQ OAB scores (p>0.05; Table 3).

**Table 3:** Comparison of ICIQ OAB scores based on different sociodemographic characteristics * A p-value <0.05 is considered statistically significant. ICIQ OAB - International Consultation on Incontinence Questionnaire Overactive Bladder

		Number of patients	Mean	Standard deviation	p-value*
Age	≤25 years	18	3.17	2.04	<0.001
	26–35 years	58	4.31	3.5	
	36–45 years	41	5.49	3.56	
	46–55 years	27	7.85	3.5	
	≥56 years	6	9.17	4.58	
Gender	Female	101	5.68	3.69	0.096
	Male	49	4.59	3.83	
Education level	No primary education	3	11	2.65	<0.001
	Primary	8	8.75	3.92	
	Middle	13	7.62	3.48	
	Secondary	42	5.64	3.75	
	Graduate	71	4.24	3.13	
	Masters or higher	13	4.54	4.33	
Smoker	No	123	5.46	3.82	0.373
	Yes	27	4.74	3.47	

A high positive and significant correlation was identified when we correlated the ICIQ UI and ICIQ OAB scores (rho=0.801, p<0.001). It was also observed that both the mean ICIQ UI and ICIQ OAB scores were significantly higher in progressive MS patients than in relapsing-remitting MS patients (p<0.05; Table 4).

**Table 4:** Comparison of ICIQ IU and ICIQ OAB scores between the two MS types * A p-value <0.05 is considered statistically significant. MS - multiple sclerosis; ICIQ UI - International Consultation on Incontinence Questionnaire-Urinary Incontinence; ICIQ OAB - International Consultation on Incontinence Questionnaire Overactive Bladder

		Number	Mean	Standard deviation	p-value*	Pearson correlation (rho)
ICIQ UI	Relapsing-remitting MS	121	5.58	5.74	0.004	rho = 0.801 (p<0.01)
	Progressive MS	29	9.31	7.479		
ICIQ OAB	Relapsing-remitting MS	121	4.9917	3.60207	0.025	
	Progressive MS	29	6.7241	4.1395		

ANCOVA models were employed to examine the influence of patient variables on ICIQ UI (Table 5) and ICIQ OAB (Table 6) scores. The marital status (F(1,135)=4.901, p=0.029, pta=0.035) and MS type (F(1,135)=7.669, p=0.006, pta=0.035) had significant influences on ICIQ IU scores. At the same time, the age (F(1,135) = 6.798, p=0.010, pta=0.048) and educational level (F(1,135)=6.918, p=0.010, pta=0.049] had significant influences on ICIQ OAB scores with a moderate effect size.

**Table 5:** ANCOVA of ICIQ UI scores for the MS patients in this study * A p-value <0.05 is considered statistically significant. df - degreed of freedom; F - analysis of variance value; ANCOVA - a univariate analysis of the covariance; ICIQ UI - International Consultation on Incontinence Questionnaire-Urinary Incontinence; MS - multiple sclerosis

Source	Type III sum of squares	df	Mean square	F	p-value*	Partial eta squared
Corrected model	1621.028	12	117.359	3.770	<0.001	0.281
Intercept	140.605	1	140.605	4.517	0.035	0.032
Age	70.916	1	70.916	2.278	0.134	0.017
Gender	18.894	1	18.894	0.607	0.437	0.004
Education	76.132	1	76.132	2.446	0.120	0.018
Smoker	50.993	1	50.993	1.638	0.203	0.012
Disease duration	8.191	1	8.191	0.263	0.609	0.002
Hypertension	26.394	1	26.394	0.848	0.359	0.006
Diabetes mellitus	9.407	1	9.407	0.302	0.583	0.002
Kidney disease	23.310	1	23.310	0.749	0.388	0.006
Cardiac disease	61.740	1	61.740	1.983	0.161	0.014
Other autoimmune disease	1.232	1	1.232	0.040	0.843	0.000
Disease-modifying therapies (DMT)	68.905	1	68.905	2.214	0.139	0.016
Type of MS	238.738	1	238.738	7.669	0.006	0.002
Error	4202.472	135	31.129			
Total	11799.000	150				
Corrected total	5845.500	149				
R squared=.281 (Adjusted R squared=0.207)_a_						

**Table 6:** ANCOVA of ICIQ OAB scores for the MS patients in this study * A p value <0.05 is considered statistically significant. df = degreed of freedom; F = analysis of variance value; ANCOVA - a univariate analysis of the covariance; ICIQ OAB - International Consultation on Incontinence Questionnaire Overactive Bladder; MS - multiple sclerosis

Source	Type III sum of squares	df	Mean square	F	p-value	Partial eta squared
Corrected model	651.655	12	46.547	4.318	<0.001	0.309
Intercept	145.059	1	145.059	13.456	<0.001	0.091
Age	73.287	1	73.287	6.798	0.010	0.048
Gender	32.821	1	32.821	3.045	0.083	0.022
Education	74.573	1	74.573	6.918	0.010	0.049
Smoker	10.168	1	10.168	.943	0.333	0.007
Disease duration	12.796	1	12.796	1.187	0.278	0.009
Hypertension	14.398	1	14.398	1.336	0.250	0.010
Diabetes mellitus	.640	1	.640	.059	0.808	0.000
Kidney disease	7.460	1	7.460	.692	0.407	0.005
Cardiac disease	17.778	1	17.778	1.649	0.201	0.012
Other autoimmune disease	5.617	1	5.617	.521	0.472	0.004
Disease-modifying therapies (DMT)	22.709	1	22.709	2.107	0.149	0.015
MS type	30.610	1	30.610	2.839	0.094	0.021
Error	1455.338	135	10.780			
Total	6363.000	150				
Corrected total	2106.993	149				
R squared=0.309 (Adjusted R squared=0.238)						

## Discussion

The prevalence of UI in MS patients ranges from 50% to 90%, making it one of the most common non-motor symptoms of the disease [8-10]. UI types observed in MS patients include detrusor overactivity (urge incontinence), stress incontinence, mixed incontinence, and overflow incontinence. The neurological damage in MS patients disrupts the bladder, spinal cord, and brain coordination, leading to impaired urinary control. Factors such as lesion location, lesion burden, disease duration, and degree of disability have been implicated in the development and severity of UI [11,12].

The findings of this study indicate that the prevalence of UI in MS patients was 64.7%, and the severity of UI was considerably higher in the advanced stages of MS, where the total neurological impairment accumulates. UI significantly impacts the QOL of individuals with MS, leading to psychological distress, social isolation, and reduced participation in daily activities [13,14]. According to Khalaf et al., the QOL was considerably lower in patients with MS who experienced immediate urgency and UI than in those who did not [15].

Our findings also revealed that patients not receiving any disease-modifying therapies (DMT) showed higher ICIQ scores. As the severity of the patient's overall UI increases, immune-modifying therapies are generally discontinued. Although various disease-modifying therapies are currently available for treating MS, there is evidence to suggest that individuals who refuse treatments at any stage of the disease, even the earliest stages, are at an increased risk of developing urinary symptoms [15].

In this study, the ICIQ UI scores were significantly higher among patients above 46 years old and who were widowed. A recent study by Abakay et al. demonstrated a strong association between older age and UI in individuals with MS [16]. The study found that as individuals with MS age, they are more likely to experience UI owing to the progressive nature of the disease and the impact it has on the nerves controlling bladder function. A study by Lin et al. reported that UI affects up to 80% of individuals with MS, with higher rates observed in older age groups [17]. These findings highlight the significant impact of older age on the development and severity of UI in individuals with MS, underscoring the need for comprehensive management strategies to address this specific issue within this population. The observed high positive correlation between ICIQ UI and ICIQ OAB scores in the context of MS underscores a significant relationship between urinary incontinence and overactive bladder symptoms. MS is fundamentally characterized by demyelination and neurodegeneration within the central nervous system. These pathophysiological changes affect neural pathways involved in bladder control. Disruptions in the communication between the brain, spinal cord, and the bladder can lead to a spectrum of urinary symptoms, encompassing both incontinence and overactive bladder manifestations [18]. The localization of MS lesions within the central nervous system plays a pivotal role. Lesions affecting areas responsible for urinary control, such as the spinal cord or regions of the brain involved in micturition reflex regulation, can directly contribute to both urinary incontinence and overactive bladder symptoms [19]. Chronic inflammation within the central nervous system not only contributes to demyelination and neuronal damage but also intensifies dysfunction in neural pathways regulating bladder function, and this could exacerbate both incontinence and overactive bladder symptoms [20]. Additionally, MS-related neurogenic bladder dysfunction can lead to a combination of symptoms, including urgency, frequency, hesitancy, incomplete emptying, and urinary leakage, which correlate with both ICIQ UI and ICIQ OAB scores [21]. Progressive MS patients may experience secondary complications like increased spasticity and muscle weakness affecting pelvic floor muscles, contributing to worsened urinary symptoms.

When individuals are asked to rate the intensity of their UI, it is not uncommon for unpleasant or upsetting symptoms to surface, some of which may be associated with social stigmas and a decrease in self-esteem. Patients with MS, for whom it is typically assumed that UI is a small concern compared to other neurological abnormalities, may be less likely to volunteer information openly and, as a result, be treated less effectively.

Our study has limitations because the participants possibly had biased perceptions of their symptoms, and objective urodynamic assessments were only available for a subset of patients and not always at the time of study enrollment. We believe that correct urodynamic diagnosis and subsequent appropriate therapy may enhance the management of urinary issues in MS patients if urodynamic tests are used more frequently.

## Conclusions

The findings of this study revealed that UI is a common and distressing symptom experienced by individuals with MS. The severity of UI symptoms was significantly higher in patients with progressive MS than in those with relapsing-remitting MS. Understanding disease prevalence, types, contributing factors, and management strategies has evolved. However, in this study, disease duration and comorbidities, such as hypertension, diabetes mellitus, kidney disease, cardiac disease, and other autoimmune diseases, were also noted. A multidisciplinary approach involving healthcare professionals from various specialties is crucial for effectively managing UI in MS patients, ultimately improving the QOL for individuals living with this chronic condition.

## Figures and Tables

**Figure 2:**
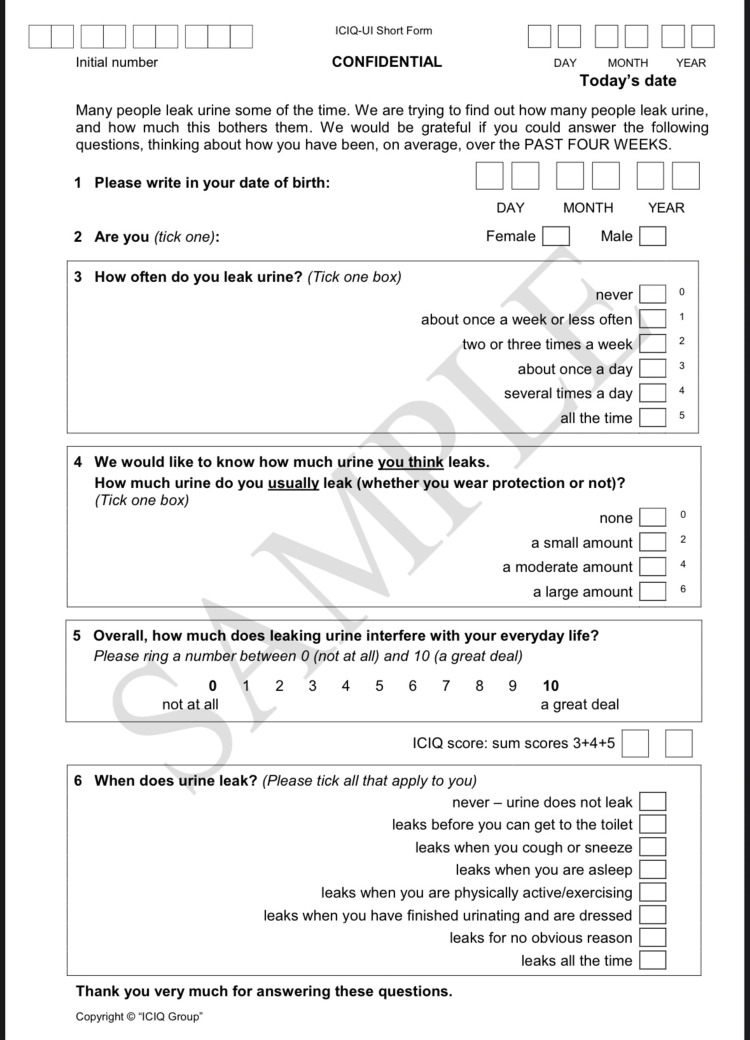
ICIQ-UI a questionnaire for evaluating the frequency, severity and impact on quality of life (QoL) of urinary incontinence in men and women in research and clinical practice across the world.

**Figure 3:**
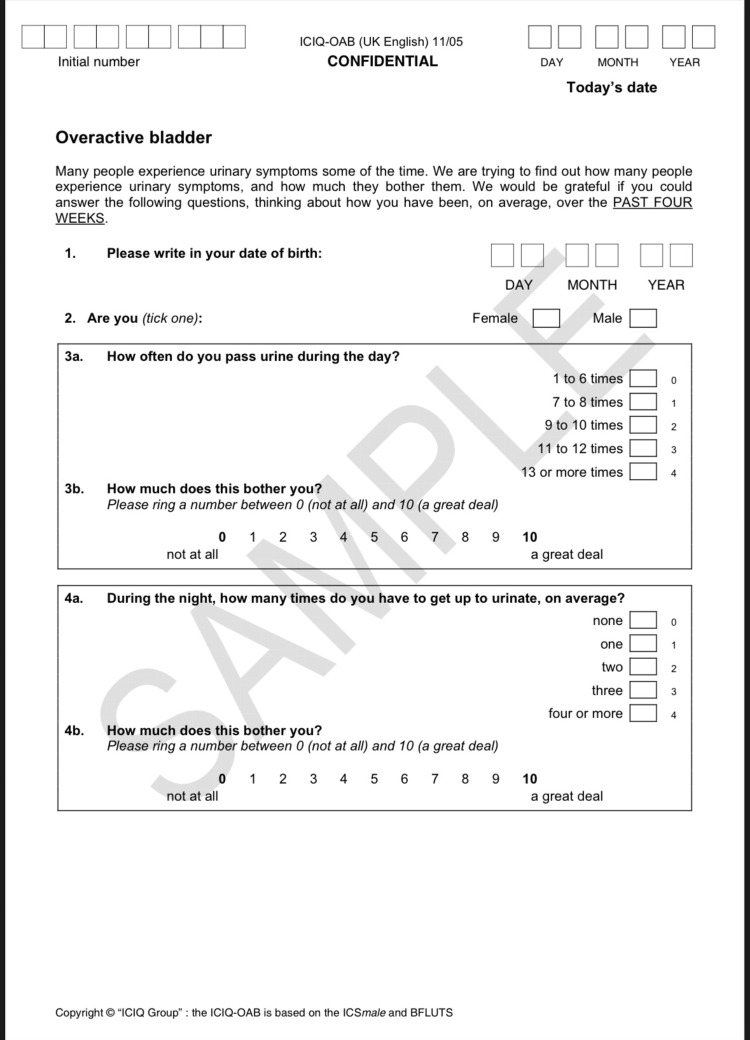
ICIQ-OAB1 questionnaire for evaluating overactive bladder and related impact on quality of life (QoL) and outcome of treatment in men and women in research and clinical practice across the world.

**Figure 4:**
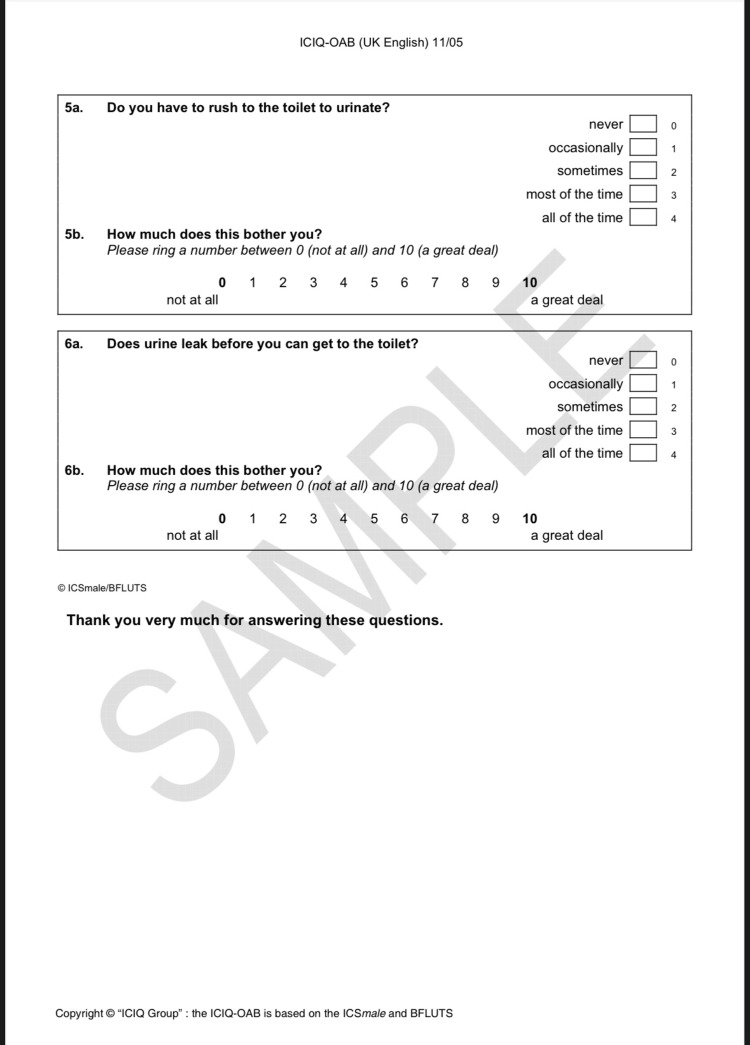
ICIQ-OAB2 questionnaire for evaluating overactive bladder and related impact on quality of life (QoL) and outcome of treatment in men and women in research and clinical practice across the world.
